# Supplementary figures and images for: Biodegradable Thermoplastic Starch/Polycaprolactone Blends with Co-Continuous Morphology Suitable for Local Release of Antibiotics
Source: Materials (Basel). 2022 Jan 30;15(3):1101. doi: 10.3390/ma15031101 (PMC8840403; doi:10.3390/ma15031101)

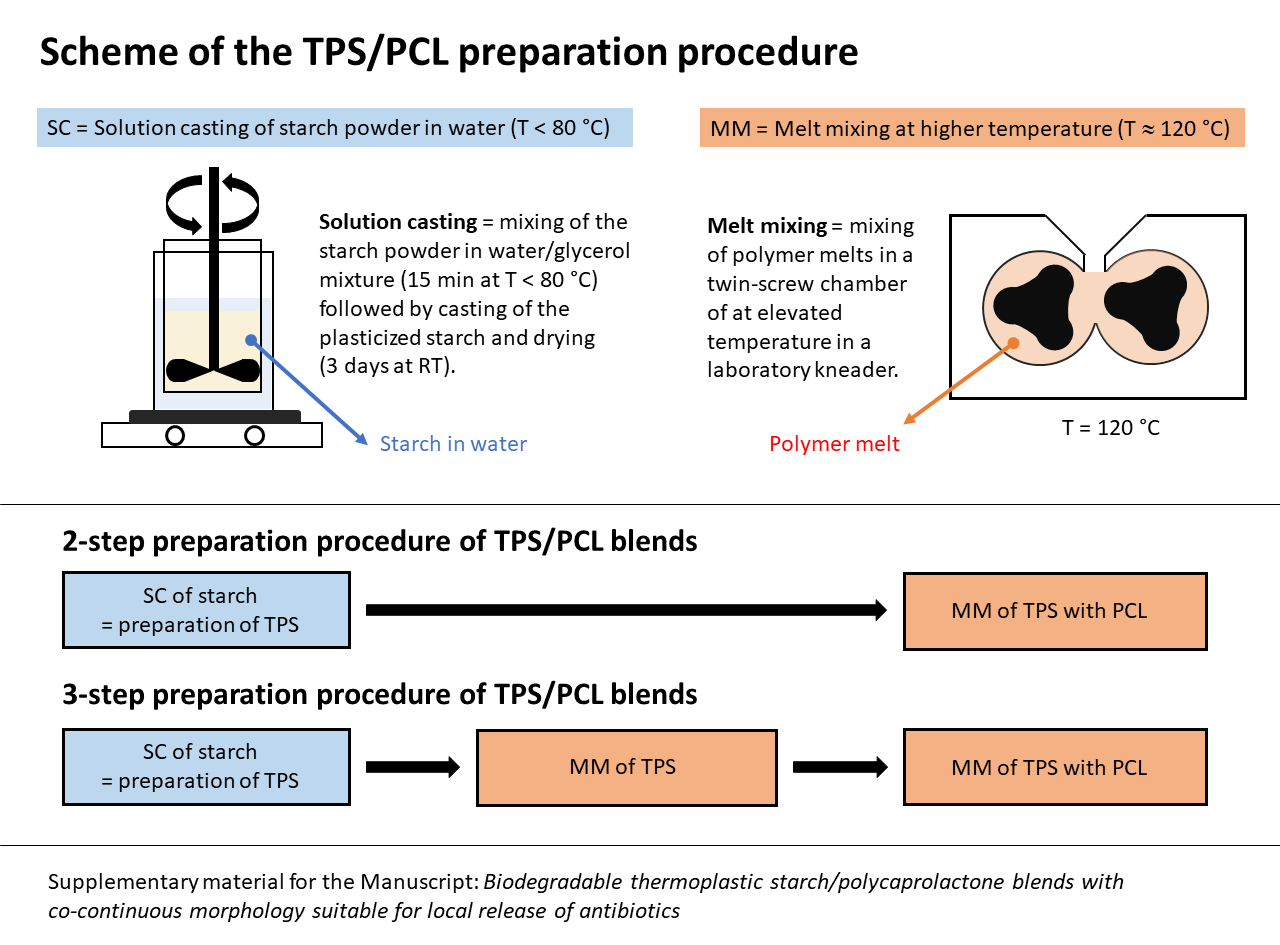

Supplement: Supplementary file 1 [file materials-15-01101-s001.zip › si2_tps-pcl_preparation.pptx.png]
